# *N*-But­oxy­carbonyl-5-oxo-l-proline ethyl ester

**DOI:** 10.1107/S1600536813007265

**Published:** 2013-03-23

**Authors:** P. Rajalakshmi, N. Srinivasan, R.V. Krishnakumar, Ibrahim Abdul Razak, Mohd Mustaqim Rosli

**Affiliations:** a Department of Physics, Thiagarajar College, Madurai 625 009, India; b X-ray Crystallography Unit, school of Physics, Universiti Sains Malaysia, 11800-USM, Penang, Malaysia

## Abstract

The mol­ecular structure of the title compound, C_12_H_19_NO_5_, may be visualized as made up of two nearly perpendicular planes [dihedral angle = 87.39 (12)°] and its crystal structure is a good example of C—H⋯O inter­actions assuming significance in optimizing supra­molecular aggregation in crystals in a mol­ecule which is severely imbalanced in terms of donors to acceptor atoms. The pyrrolidine ring adopts a (^3^ *T*_2_) twist conformation with puckering parameters *Q* = 0.2630 (4) Å and ϕ = 59 (9)°. The crystal structure features *R*_2_^4^(10) and *R*_3_^4^(26) ring motifs formed by four weak C—H⋯O inter­actions, leading to supra­molecular sheets lying parallel to the *bc* plane.

## Related literature

For general background, see: Holladay *et al.* (1991 ▶); Kayushina & Vainshtein (1966 ▶); Wu (2009 ▶). For the biological activity of proline derivatives, see: Hayashi *et al.* (2003 ▶); Nishikawa & Murakami (2005 ▶). For hydrogen bonding, see: Bernstein *et al.* (1995 ▶). For puckering parameters, see: Cremer & Pople (1975 ▶). For a description of the Cambridge Structural Database, see: Allen (2002 ▶).
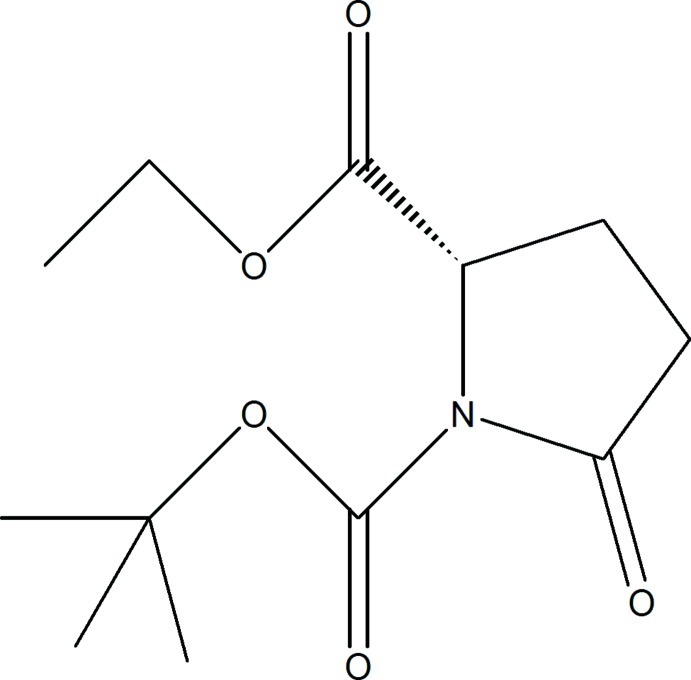


## Experimental

### 

#### Crystal data

C_12_H_19_NO_5_
*M* *_r_* = 257.28
Orthorhombic, 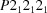

*a* = 26.6884 (13) Å
*b* = 5.7650 (3) Å
*c* = 8.7054 (4) Å
*V* = 1339.40 (11) Å^3^
*Z* = 4
Cu *K*α radiation
μ = 0.83 mm^−1^
*T* = 100 K
0.44 × 0.21 × 0.11 mm

#### Data collection

Bruker SMART APEXII CCD area-detector diffractometer
Absorption correction: multi-scan (*SADABS*; Bruker, 2009 ▶) *T*_min_ = 0.711, *T*_max_ = 0.914
9641 measured reflections
2184 independent reflections
2144 reflections with *I* > 2σ(*I*)
*R*_int_ = 0.049

#### Refinement

*R*[*F* ^2^ > 2σ(*F* ^2^)] = 0.060
*wR*(*F* ^2^) = 0.166
*S* = 1.09
2184 reflections
167 parameters
H-atom parameters constrained
Δρ_max_ = 0.37 e Å^−3^
Δρ_min_ = −0.22 e Å^−3^

### 

Data collection: *APEX2* (Bruker, 2009 ▶); cell refinement: *SAINT* (Bruker, 2009 ▶); data reduction: *SAINT*; program(s) used to solve structure: *SHELXTL* (Sheldrick, 2008 ▶); program(s) used to refine structure: *SHELXTL*; molecular graphics: *SHELXTL*; software used to prepare material for publication: *SHELXTL* and *PLATON* (Spek, 2009 ▶).

## Supplementary Material

Crystal structure: contains datablock(s) I, global. DOI: 10.1107/S1600536813007265/bx2436sup1.cif

Structure factors: contains datablock(s) I. DOI: 10.1107/S1600536813007265/bx2436Isup2.hkl

Supplementary material file. DOI: 10.1107/S1600536813007265/bx2436Isup3.cml

Additional supplementary materials:  crystallographic information; 3D view; checkCIF report

## Figures and Tables

**Table 1:** Hydrogen-bond geometry (Å, °)

*D*—H⋯*A*	*D*—H	H⋯*A*	*D*⋯*A*	*D*—H⋯*A*
C2—H2*A*⋯O3^i^	1.00	2.51	3.436 (5)	154
C3—H3*B*⋯O3^ii^	0.99	2.46	3.095 (5)	121
C6—H6*B*⋯O5^iii^	0.99	2.50	3.344 (5)	143
C12—H12*A*⋯O5^i^	0.98	2.55	3.327 (5)	136

Symmetry codes: (i) 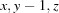; (ii) 
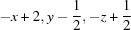
; (iii) 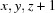.

**Fig. 1.**
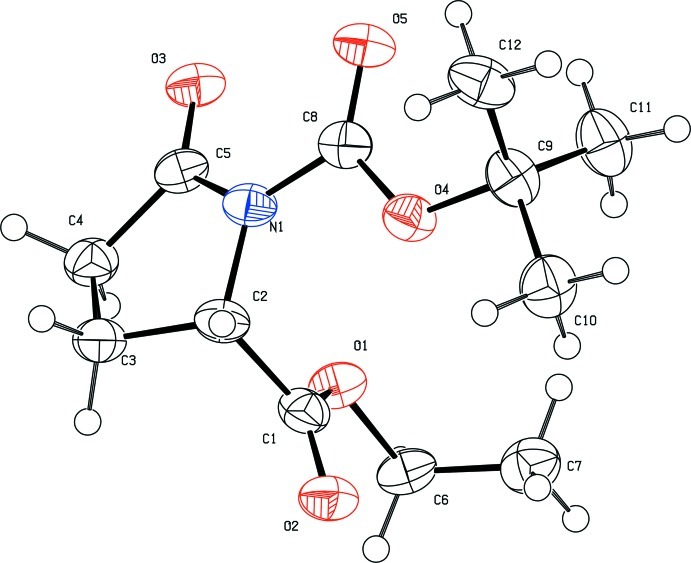
The molecule of title compound, showing the atom-labeling scheme. Displacement ellipsoids are drawn at the 50% probability.

**Fig. 2.**
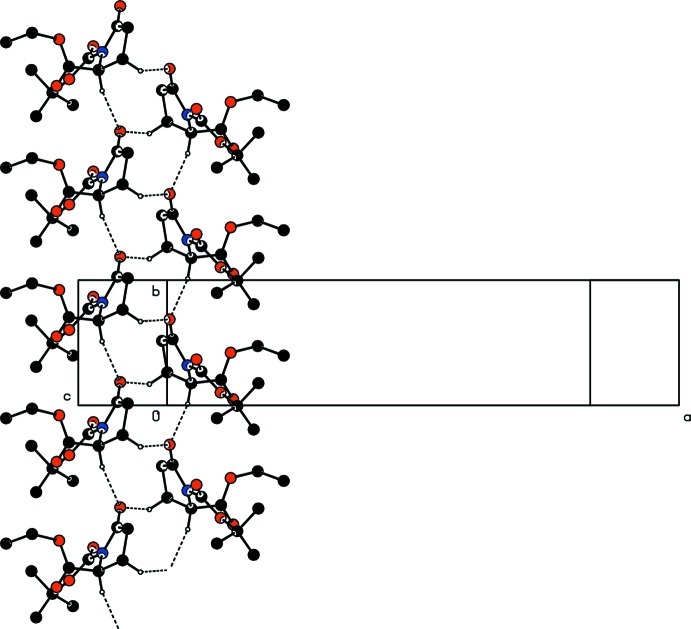
Part of the crystal structure of title compound, showing the formation of a R42(10) chain running parallel to the b-axis. Dashed lines indicate hydrogen bonds. For the sake of clarity, H atoms are omitted.

**Fig. 3.**
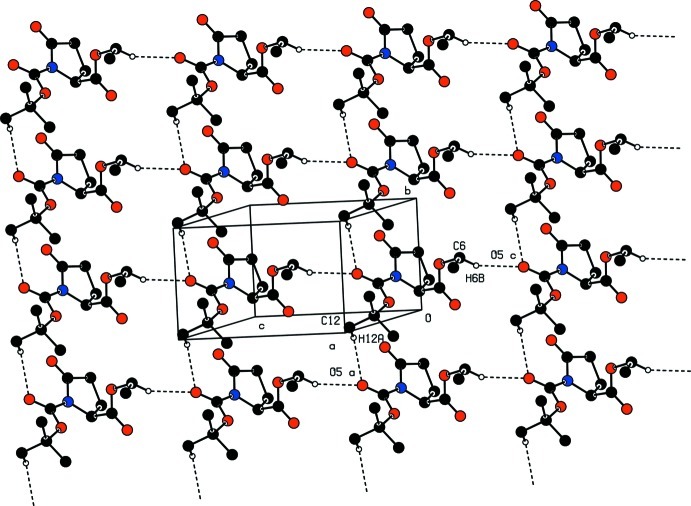
Part of the crystal structure of title compound, showing the formation of supramolecular sheets of R43(26) ring parallel to the bc-plane. Dashed lines indicate hydrogen bonds. For the sake of clarity, H atoms bonded to C atoms have been omitted

## supplementary crystallographic information

### Comment

Proline (Kayushina & Vainshtein, 1966) is a functional amino acid that participates in the regulation of key metabolic pathways essential for maintenance, growth, reproduction and immunity (Wu, 2009). Many substituted proline derivatives are known for their active role in biological functions. For instance, 5-oxo-proline or pyroglutamic acid, found in many proteins including bacteriorhodopsin, acts as a proton pump, captures light energy and uses it to move protons across the membrane out of the cell (Hayashi, *et al.*, 2003; Nishikawa, *et al.*, 2005). Also, N-boc-4-oxo-*L*-proline ethyl ester is a part of the starting material on stereoselective synthesis of peptide hormone cholecystokinin (Holladay, *et al.*, 1991). The present paper describes the accurate description of the crystal structures of N-boc-5-oxo-*L*-proline ethyl ester (Fig.1).

The molecular mainframe of the title compound may be visualized as made up of two nearly perpendicular planes (N1/C8/O4/O5) and (O1/C1/O2/C2) with 87.39 (12) °. The orientation of the carbonyl O3 (substituted to the pyrrolidine ring), O4 (ethyl ester carbonyl) and O5 (*tert*-butyloxycarbonyl) may be described by the torsion angles about the C2—N1 bond which are -171.05 (4) °, -54.37 (6) ° and -170.82 (4) °, respectively. The pyrrolidine adopts the *twisted* conformation (^3^T_2_) with C2 and C3 atoms deviating from the plane defined by the rest of the atoms by about -0.3872 (6) Å and 0.4068 (6) Å, respectively with the associated puckering parameters (Cremer & Pople, 1975) of Q=0.2630 (4) Å, φ= 59 (9) °.

The crystal structure of the title compound is a simple example which demonstrates the importance of weak C–H···O interactions assuming significance in optimizing supramolecular aggregation in crystals. The molecule may be thought of as highly imbalanced in terms of donors to acceptor atoms which has at least three carbonyl O atoms O3, O4 and O5, out of the total five O atoms (O1→O5), available for participation in intermolecular interactions. O1 and O2 of the respective ethyl ester and the *tert*-butyloxy groups do not participate in the hydrogen-bonding environment owing to unfavourable steric reasons. The intermolecular interaction patterns may be vizualized as molecular chains interconnected to each other to form a sheet. The ethyl C2 and ethyl C3 atoms act as donors to the carbonyl O3 which is a bifurcated acceptor at (*x, y - 1, z*) and (-*x* + 2, *y* - 1/2, -*z* + 1/2), respectively. The associated graph-set motif (Bernstein *et al.*, 1995) is a *R*^4^_2_(10) ring through C2—H2A···O3 and C3—H3A···O3 hydrogen bond leading to chains parallel to the *b*-axis (Fig.2). The ethyl C6 and ethyl C12 atoms act as donors to the bifurcated acceptor (carbonyl) O5 at (*x, y, z + 1*) and (*x, y - 1, z*), respectively, forming a *R*^4^_3_ (26) ring motif through a C6—H6B···O5 and C12—H12A···O5 hydrogen bonds forming sheets parallel to the *bc*-plane (Fig.3).

### Experimental

To a solution of oxo proline ethylester (0.5 g,3.26 mmol) in dichloromethane (10 ml), was added triethylamine (0.5 ml, 3.26 mmol), di-*tert*-butyl-dicarbonate (1.4 g, 6.52 mmol) and 4-(dimethylamino)-pyridine (0.4 g, 3.26 mmol) under N2. The resulting yellow solution was stirred at room temperature for 2 h. The reaction mixture was concentrated. The residue was purified by column chromatography to afford boc-oxo-*L*-proline ethylester (0.8 g, 95%). Crystals of the title compound were grown from its solution in ethanol by slow evaporation at room temperature.

### Refinement

All the hydrogen atoms were placed at geometrically calculated positions. They were allowed to ride on respective parent atoms with *U*_iso_ values constrained to 1.2 times *U*_eq_ (1.5 times for ethyl H atoms) and the target C—H distance fixed at 0.96 Å for ethyl hydrogen atoms and 0.93 Å for all others.

### Crystal data

**Table:**

C_12_H_19_NO_5_	*F*(000) = 552
*M**_r_* = 257.28	*D*_x_ = 1.276 Mg m^−^^3^
Orthorhombic, *P*2_1_2_1_2_1_	Cu *K*α radiation, λ = 1.54178 Å
Hall symbol: P 2ac 2ab	Cell parameters from 2145 reflections
*a* = 26.6884 (13) Å	θ = 5.2–67.7°
*b* = 5.7650 (3) Å	µ = 0.83 mm^−^^1^
*c* = 8.7054 (4) Å	*T* = 100 K
*V* = 1339.40 (11) Å^3^	Block, colourless
*Z* = 4	0.44 × 0.21 × 0.11 mm

### Data collection

**Table:**

Bruker SMART APEXII CCD area-detector diffractometer	2184 independent reflections
Radiation source: fine-focus sealed tube	2144 reflections with *I* > 2σ(*I*)
Graphite monochromator	*R*_int_ = 0.049
φ and ω scans	θ_max_ = 65.0°, θ_min_ = 7.1°
Absorption correction: multi-scan (*SADABS*; Bruker, 2009)	*h* = −31→31
*T*_min_ = 0.711, *T*_max_ = 0.914	*k* = −5→6
9641 measured reflections	*l* = −10→10

### Refinement

**Table:**

Refinement on *F*^2^	Primary atom site location: structure-invariant direct methods
Least-squares matrix: full	Secondary atom site location: difference Fourier map
*R*[*F*^2^ > 2σ(*F*^2^)] = 0.060	Hydrogen site location: inferred from neighbouring sites
*wR*(*F*^2^) = 0.166	H-atom parameters constrained
*S* = 1.09	*w* = 1/[σ^2^(*F*_o_^2^) + (0.0688*P*)^2^ + 1.9721*P*] where *P* = (*F*_o_^2^ + 2*F*_c_^2^)/3
2184 reflections	(Δ/σ)_max_ < 0.001
167 parameters	Δρ_max_ = 0.37 e Å^−^^3^
0 restraints	Δρ_min_ = −0.22 e Å^−^^3^

### Special details

**Table:**

Geometry. All e.s.d.'s (except the e.s.d. in the dihedral angle between two l.s. planes) are estimated using the full covariance matrix. The cell e.s.d.'s are taken into account individually in the estimation of e.s.d.'s in distances, angles and torsion angles; correlations between e.s.d.'s in cell parameters are only used when they are defined by crystal symmetry. An approximate (isotropic) treatment of cell e.s.d.'s is used for estimating e.s.d.'s involving l.s. planes.
Refinement. Refinement of *F*^2^ against ALL reflections. The weighted *R*-factor *wR* and goodness of fit *S* are based on *F*^2^, conventional *R*-factors *R* are based on *F*, with *F* set to zero for negative *F*^2^. The threshold expression of *F*^2^ > σ(*F*^2^) is used only for calculating *R*-factors(gt) *etc*. and is not relevant to the choice of reflections for refinement. *R*-factors based on *F*^2^ are statistically about twice as large as those based on *F*, and *R*- factors based on ALL data will be even larger.

### Fractional atomic coordinates and isotropic or equivalent isotropic displacement parameters (Å^2^)

**Table:**

	*x*	*y*	*z*	*U*_iso_*/*U*_eq_	
O1	0.90408 (10)	0.9206 (5)	0.6629 (3)	0.0370 (7)	
O4	0.87203 (9)	0.6029 (5)	0.3651 (3)	0.0363 (6)	
O3	0.95830 (10)	1.1864 (5)	0.2879 (3)	0.0385 (6)	
O2	0.91287 (10)	0.5496 (5)	0.7415 (3)	0.0418 (7)	
O5	0.88646 (10)	0.8660 (5)	0.1762 (3)	0.0390 (7)	
N1	0.93969 (11)	0.8201 (6)	0.3819 (3)	0.0322 (7)	
C5	0.96730 (14)	1.0270 (7)	0.3731 (4)	0.0336 (8)	
C4	1.00831 (15)	1.0151 (7)	0.4918 (4)	0.0386 (9)	
H4A	1.0015	1.1235	0.5774	0.046*	
H4B	1.0411	1.0549	0.4456	0.046*	
C3	1.00806 (15)	0.7635 (7)	0.5485 (4)	0.0378 (9)	
H3A	1.0148	0.7561	0.6603	0.045*	
H3B	1.0335	0.6700	0.4938	0.045*	
C2	0.95504 (14)	0.6769 (7)	0.5120 (4)	0.0346 (8)	
H2A	0.9563	0.5103	0.4803	0.042*	
C1	0.92075 (14)	0.7041 (7)	0.6499 (4)	0.0362 (9)	
C6	0.87505 (15)	0.9735 (8)	0.8004 (4)	0.0412 (9)	
H6A	0.8760	1.1427	0.8195	0.049*	
H6B	0.8904	0.8948	0.8898	0.049*	
C7	0.82162 (16)	0.8971 (8)	0.7847 (5)	0.0473 (10)	
H7A	0.8025	0.9492	0.8744	0.071*	
H7B	0.8203	0.7276	0.7781	0.071*	
H7C	0.8072	0.9647	0.6915	0.071*	
C8	0.89734 (14)	0.7707 (7)	0.2939 (4)	0.0351 (9)	
C9	0.82710 (14)	0.4960 (7)	0.2939 (5)	0.0414 (9)	
C10	0.81499 (16)	0.3074 (8)	0.4085 (6)	0.0484 (11)	
H10A	0.8427	0.1963	0.4126	0.073*	
H10B	0.7843	0.2271	0.3769	0.073*	
H10C	0.8101	0.3764	0.5102	0.073*	
C11	0.78525 (15)	0.6769 (8)	0.2898 (6)	0.0510 (11)	
H11A	0.7814	0.7463	0.3920	0.077*	
H11B	0.7538	0.6023	0.2597	0.077*	
H11C	0.7937	0.7981	0.2153	0.077*	
C12	0.83972 (17)	0.3976 (8)	0.1365 (5)	0.0483 (11)	
H12A	0.8695	0.2986	0.1444	0.072*	
H12B	0.8464	0.5251	0.0650	0.072*	
H12C	0.8114	0.3056	0.0987	0.072*	

### Atomic displacement parameters (Å^2^)

**Table:**

	*U*^11^	*U*^22^	*U*^33^	*U*^12^	*U*^13^	*U*^23^
O1	0.0497 (15)	0.0307 (15)	0.0307 (14)	−0.0008 (11)	0.0045 (11)	0.0004 (11)
O4	0.0413 (14)	0.0347 (15)	0.0327 (14)	−0.0024 (11)	−0.0049 (11)	0.0012 (11)
O3	0.0514 (15)	0.0343 (15)	0.0299 (13)	−0.0023 (12)	0.0013 (11)	0.0055 (13)
O2	0.0514 (16)	0.0403 (17)	0.0337 (14)	−0.0004 (12)	−0.0029 (12)	0.0086 (13)
O5	0.0558 (16)	0.0319 (15)	0.0292 (14)	0.0016 (12)	−0.0065 (11)	0.0035 (11)
N1	0.0416 (16)	0.0317 (17)	0.0233 (14)	0.0021 (13)	−0.0015 (13)	−0.0043 (14)
C5	0.043 (2)	0.033 (2)	0.0250 (17)	0.0001 (16)	0.0050 (15)	−0.0017 (17)
C4	0.044 (2)	0.042 (2)	0.0294 (19)	−0.0046 (18)	0.0010 (16)	−0.0040 (18)
C3	0.042 (2)	0.043 (2)	0.0288 (19)	0.0037 (17)	−0.0004 (15)	0.0043 (17)
C2	0.044 (2)	0.029 (2)	0.0311 (18)	0.0025 (16)	−0.0027 (15)	0.0054 (16)
C1	0.0379 (19)	0.041 (2)	0.0293 (19)	−0.0021 (16)	−0.0072 (15)	−0.0011 (18)
C6	0.051 (2)	0.045 (2)	0.0272 (18)	0.0005 (18)	0.0077 (17)	−0.0008 (18)
C7	0.052 (2)	0.046 (3)	0.044 (2)	0.0044 (19)	0.0069 (19)	−0.002 (2)
C8	0.041 (2)	0.030 (2)	0.034 (2)	0.0048 (15)	0.0007 (16)	0.0002 (16)
C9	0.041 (2)	0.034 (2)	0.048 (2)	−0.0004 (17)	−0.0083 (17)	−0.005 (2)
C10	0.048 (2)	0.037 (3)	0.061 (3)	−0.0058 (18)	−0.001 (2)	0.004 (2)
C11	0.044 (2)	0.040 (3)	0.069 (3)	0.0004 (18)	−0.011 (2)	−0.003 (3)
C12	0.064 (3)	0.037 (2)	0.044 (2)	−0.002 (2)	−0.015 (2)	−0.003 (2)

### Geometric parameters (Å, º)

**Table:**

O1—C1	1.330 (5)	C6—C7	1.499 (6)
O1—C6	1.458 (4)	C6—H6A	0.9900
O4—C8	1.332 (5)	C6—H6B	0.9900
O4—C9	1.484 (5)	C7—H7A	0.9800
O3—C5	1.205 (5)	C7—H7B	0.9800
O2—C1	1.214 (5)	C7—H7C	0.9800
O5—C8	1.198 (5)	C9—C10	1.510 (6)
N1—C8	1.395 (5)	C9—C12	1.521 (6)
N1—C5	1.404 (5)	C9—C11	1.529 (6)
N1—C2	1.461 (5)	C10—H10A	0.9800
C5—C4	1.506 (5)	C10—H10B	0.9800
C4—C3	1.533 (6)	C10—H10C	0.9800
C4—H4A	0.9900	C11—H11A	0.9800
C4—H4B	0.9900	C11—H11B	0.9800
C3—C2	1.534 (5)	C11—H11C	0.9800
C3—H3A	0.9900	C12—H12A	0.9800
C3—H3B	0.9900	C12—H12B	0.9800
C2—C1	1.517 (5)	C12—H12C	0.9800
C2—H2A	1.0000		
			
C1—O1—C6	116.4 (3)	H6A—C6—H6B	107.9
C8—O4—C9	121.1 (3)	C6—C7—H7A	109.5
C8—N1—C5	124.7 (3)	C6—C7—H7B	109.5
C8—N1—C2	122.5 (3)	H7A—C7—H7B	109.5
C5—N1—C2	112.0 (3)	C6—C7—H7C	109.5
O3—C5—N1	125.2 (3)	H7A—C7—H7C	109.5
O3—C5—C4	127.0 (4)	H7B—C7—H7C	109.5
N1—C5—C4	107.8 (3)	O5—C8—O4	127.4 (4)
C5—C4—C3	105.1 (3)	O5—C8—N1	124.9 (4)
C5—C4—H4A	110.7	O4—C8—N1	107.7 (3)
C3—C4—H4A	110.7	O4—C9—C10	101.3 (3)
C5—C4—H4B	110.7	O4—C9—C12	110.6 (3)
C3—C4—H4B	110.7	C10—C9—C12	111.9 (4)
H4A—C4—H4B	108.8	O4—C9—C11	108.5 (3)
C4—C3—C2	104.2 (3)	C10—C9—C11	110.5 (4)
C4—C3—H3A	110.9	C12—C9—C11	113.3 (4)
C2—C3—H3A	110.9	C9—C10—H10A	109.5
C4—C3—H3B	110.9	C9—C10—H10B	109.5
C2—C3—H3B	110.9	H10A—C10—H10B	109.5
H3A—C3—H3B	108.9	C9—C10—H10C	109.5
N1—C2—C1	112.7 (3)	H10A—C10—H10C	109.5
N1—C2—C3	103.6 (3)	H10B—C10—H10C	109.5
C1—C2—C3	111.0 (3)	C9—C11—H11A	109.5
N1—C2—H2A	109.8	C9—C11—H11B	109.5
C1—C2—H2A	109.8	H11A—C11—H11B	109.5
C3—C2—H2A	109.8	C9—C11—H11C	109.5
O2—C1—O1	125.1 (4)	H11A—C11—H11C	109.5
O2—C1—C2	123.3 (4)	H11B—C11—H11C	109.5
O1—C1—C2	111.5 (3)	C9—C12—H12A	109.5
O1—C6—C7	111.7 (3)	C9—C12—H12B	109.5
O1—C6—H6A	109.3	H12A—C12—H12B	109.5
C7—C6—H6A	109.3	C9—C12—H12C	109.5
O1—C6—H6B	109.3	H12A—C12—H12C	109.5
C7—C6—H6B	109.3	H12B—C12—H12C	109.5
			
C8—N1—C5—O3	−0.8 (6)	N1—C2—C1—O2	149.4 (4)
C2—N1—C5—O3	−171.0 (4)	C3—C2—C1—O2	−94.8 (4)
C8—N1—C5—C4	177.3 (3)	N1—C2—C1—O1	−35.4 (4)
C2—N1—C5—C4	7.1 (4)	C3—C2—C1—O1	80.3 (4)
O3—C5—C4—C3	−171.4 (4)	C1—O1—C6—C7	−81.1 (4)
N1—C5—C4—C3	10.6 (4)	C9—O4—C8—O5	5.8 (6)
C5—C4—C3—C2	−23.0 (4)	C9—O4—C8—N1	−174.7 (3)
C8—N1—C2—C1	−72.0 (4)	C5—N1—C8—O5	19.8 (6)
C5—N1—C2—C1	98.5 (4)	C2—N1—C8—O5	−170.9 (4)
C8—N1—C2—C3	167.9 (3)	C5—N1—C8—O4	−159.7 (3)
C5—N1—C2—C3	−21.6 (4)	C2—N1—C8—O4	9.5 (5)
C4—C3—C2—N1	26.7 (4)	C8—O4—C9—C10	175.3 (3)
C4—C3—C2—C1	−94.6 (4)	C8—O4—C9—C12	56.5 (5)
C6—O1—C1—O2	1.3 (5)	C8—O4—C9—C11	−68.4 (4)
C6—O1—C1—C2	−173.7 (3)		

### Hydrogen-bond geometry (Å, º)

**Table:**

*D*—H···*A*	*D*—H	H···*A*	*D*···*A*	*D*—H···*A*
C2—H2*A*···O3^i^	1.00	2.51	3.436 (5)	154
C3—H3*B*···O3^ii^	0.99	2.46	3.095 (5)	121
C6—H6*B*···O5^iii^	0.99	2.50	3.344 (5)	143
C12—H12*A*···O5^i^	0.98	2.55	3.327 (5)	136

Symmetry codes: (i) *x*, *y*−1, *z*; (ii) −*x*+2, *y*−1/2, −*z*+1/2; (iii) *x*, *y*, *z*+1.
